# Microglia senescence is related to neuropathic pain–associated comorbidities in the spared nerve injury model

**DOI:** 10.1097/j.pain.0000000000002807

**Published:** 2022-10-19

**Authors:** Vittoria Borgonetti, Nicoletta Galeotti

**Affiliations:** Department of Neuroscience, Psychology, Drug Research and Child Health (NEUROFARBA), Section of Pharmacology and Toxicology, University of Florence, Florence, Italy

**Keywords:** Neuroinflammation, Microglia, Neuropathic pain, Senescence, Mood disorders, Cognitive impairment

## Abstract

The increased presence of senescent cells in different neurological diseases suggests the contribution of senescence in the pathophysiology of neurodegenerative disorders. Microglia can adapt to any type of disturbance of the homeostasis of the central nervous system, and its altered activity can lead to permanent and unresolvable damage. The aim of this work was to characterize the behavioural phenotype of spared nerve injury mice and then associate it with senescence-related mechanisms. In this work, we investigated the timing of the onset of anxiety, depression, or memory decline associated with peripheral neuropathic pain and their correlation with the presence of microglial cellular senescence. Spared nerve injury mice showed a persistent pain hypersensitivity from 3 days after surgery. Twenty-eight days after nerve injury, they also developed anxiety, depression, and cognitive impairment. The appearance of these symptoms was coincident to a significant increase of senescence markers, such as β-galactosidase and senescent-associated secretory phenotype, at the microglial level in the spinal cord and hippocampus of spared nerve injury animals. These markers were unaltered at previous time points. In murine immortalized microglial cells (BV2) stimulated with LPS 500 ng/mL for 10 days (4 hours/day) every other day, we observed an increase of β-galactosidase and senescent-associated secretory phenotype appearance, a reduction of cell viability, and an increase of senescence-associated heterochromatin foci. Therefore, present findings could represent an important step to a better understanding of the pathophysiological cellular mechanisms in comorbidities related to neuropathic pain states.

## Abstract 2

Supplemental Digital Content is Available in the Text.

Detection of senescent microglia in animals with neuropathy. Optimization of an in vitro model of microglial senescence.

## 1. Introduction

The management of neuropathic pain (NP) is a relevant clinical problem because of the poor responsiveness of patients to available analgesic drugs.^12^ In addition, anxiety, depression, cognitive impairment, and other mood disorders are comorbidities that characterize approximately 34% of patients with neuropathic pain. The onset of these comorbidities can drastically worsen the patients' quality of life, exacerbating painful conditions. However, there are no effective and safe treatments able to manage these comorbidities.^31,38,46,49^

The contribution of spinal microglia in the onset and progression of NP is well established,^28,47^ but the role of microglia in supraspinal structures during neuropathies is less understood. Recent studies showed that trauma-induced neuropathic pain conditions provoke the activation of microglia in several brain areas, such as the hippocampus, amygdala, thalamus, and anterior cingulate cortex, that contribute to the NP-associated sensory and emotional disorders.^30,42^

Microglia can adapt to any type of disturbance of the homeostasis of the central nervous system (CNS), and their lack of activity can lead to permanent and unresolvable damage.^3^ Uncontrolled increases in microglial cell activity result in the loss of normal physiological microglial functionality, with the insurgence of microglial senescence. An increased presence of senescent cells in different neurological diseases suggests the contribution of senescence in the pathophysiology of these disorders.^2^ Indeed, microglial aging may actively contribute to the development of neurodegenerative diseases, including Alzheimer disease or Parkinson disease, because of reduced neuroprotective function, increased neurotoxicity, and altered responses to stimuli.^32^

The recent progress in the development of animal models has increased the number of studies focusing on the mechanisms underlying the comorbidities of chronic pain, such as depression and anxiety.^22^ However, targets capable of modulating both pain and emotional symptoms have not been found yet. Neuropathic pain starts with an inflammatory process, which, if unresolved, can lead to alteration of the normal activity of the CNS environment. In this condition, microglia may not withstand this continuous stimulus and lose their function until become senescent. Hence, in this work, we investigated the presence of microglial senescent cells at spinal and supraspinal levels in an animal model of NP, the spared nerve injury (SNI). The timing of the onset of pain hypersensitivity and of various comorbidities associated with peripheral NP in mice was defined, and a correlation between microglial cellular senescence and NP-associated symptoms was assessed.

## 2. Materials and Methods

### 2.1. Animals

CD1 male mice (2 months) weighting approximately 20 g (Envigo, Varese, Italy) were housed in the Ce.S.A.L. (Centro Stabulazione Animali da Laboratorio, University of Florence) vivarium and used 3 days after their arrival. Mice were housed in standard cages, kept at 23 ± 1°C with a 12-hour light/dark cycle, light on at 7 am, and fed with standard laboratory diet and tap water ad libitum. All tests were conducted during the light phase. The experimental protocol was approved by the Institution's Animal Care and Research Ethics Committee (University of Florence, Italy), under license from the Italian Department of Health (54/2014-B). Mice were treated in accordance with the relevant European Union (Directive 2010/63/EU, the council of September 22, 2010, on the protection of animals used for scientific purposes) and international regulations (Guide for the Care and Use of Laboratory Animals, US National Research Council, 2011). All studies involving animals are reported in accordance with the ARRIVE 2.0 and British Journal of Pharmacology guidelines for experiments involving animals.^29,43^ The experimental protocol was designed to minimize the number of animals used and their suffering. The G power software was used to perform a power analysis to choose the number of animals per experiment.^11^

A schematic representation of the animal procedure has been reported in Table 1.

**Table 1:** Schematic representation of spared nerve injury animals' model and test.

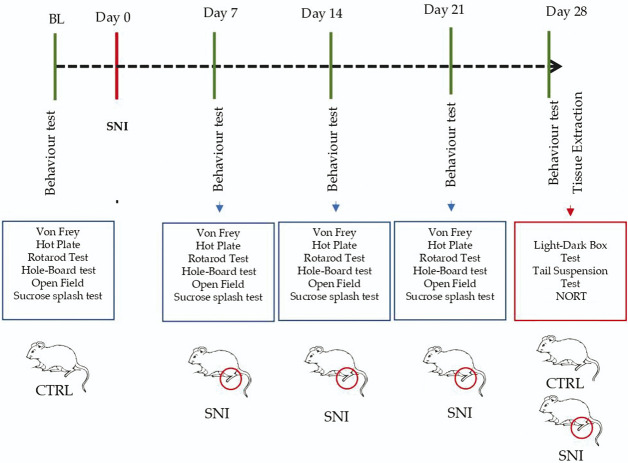

BL, baseline; CTRL, control; NORT, novel object recognition test; SNI, spared nerve injury.

### 2.2. Spared nerve injury procedure and von Frey filaments

The SNI model represents a peripheral mononeuropathy model that was performed as previously described.^4^ Briefly, the animals were anaesthetized with 4% isoflurane in O_2_/N_2_O (30:70 vol/vol). The right paw, conventionally called “ipsi,” was operated, whereas the left “contra” remained intact. A skin incision was made laterally on the right limb to identify the sciatic nerve. Of the 3 branches of the nerve, the common peroneal and tibial were tied together with 5.0 silk suture thread (Ethicon; Johnson & Johnson Intl, Brussels, Belgium) and cut, with the sural remaining intact. Tests were then conducted 3 days after the operation to detect the postsurgical appearance of mechanical allodynia and thermal hyperalgesia. The experimental groups were control (CTRL) mice (n = 21; sham-operated mice) and SNI mice (n = 25; operated mice) subdivided into 2 different cohorts of animals. Sex differences in response to pain have been described in the SNI model in which microgliosis and pain hypersensitivity were mainly detected in male mice.^19^ Rosmarinic acid (RA; Merck, Darmstadt, Germany), used as the senomorphic reference drug,^6^ was solubilized in carboxymethylcellulose 1% (CMC1%) at the dose of 5 mg/kg and orally administered.

### 2.3. Allodynia and hyperalgesia

#### 2.3.1. Mechanical allodynia measured with von Frey filaments

Animals were placed in Plexiglas trays (8.5 × 3.4 × 3.4 [h] cm) and allowed to adapt for approximately 1 hour. Mechanical allodynia was measured using von Frey monofilaments using the up–down protocol, which are used with increasing strengths from 0.07, 0.16, 0.4, 0.6, 1.0, and 1.4 up to 2.0 g. These filaments have been applied in both the operated (ipsilateral) and nonoperated (contralateral) paw. A response is considered positive when animals respond to a filament 3 times out of 5 repetitive stimuli. In case of a negative response, the next filament was applied. This test was performed in the baseline (BL) condition and on day 7, 14, and 21 after surgery.^5,15^

#### 2.3.2. Thermal hyperalgesia measured with the hot-plate test

To assess thermal hyperalgesia, the hot-plate test was used, which consists of measuring the animal's response time to a thermal stimulus (52°C) applied to a 24-cm-diameter electrically operated device. Mice were placed on the hot plate, surrounded by a transparent acrylic cage. A response is considered positive when the animals lick themselves, shake their heads, or jump. This test was performed in the BL condition and on day 7, 14, and 21 after surgery.^10^

### 2.4. Locomotor activity

#### 2.4.1. Rotarod test

The possible onset of motor side effects induced by treatment was evaluated with the rotarod test, as previously described.^9^ Animals have been habituated before starting the test. The rotarod is an instrument consisting of a rotating rod with a diameter of approximately 5 cm. This rod is placed at a height of about 15 cm from the base of the instrument. The speed of the rod was 16 rpm, and the time in which the number of falls of the animal was recorded was 30 seconds. This test was performed in the BL condition and on day 7, 14, and 21 after surgery.

#### 2.4.2. Hole-board test

The hole-board test is commonly used to verify the effect of a drug on the spontaneous mobility and exploratory activity.^9^ In this test, animals are observed for 5 minutes and kept on a square board (50 × 50 × 15 cm). Four electro photocells placed on each side record the animal's activity on the board (which we define as spontaneous mobility). On this board, there are holes with a diameter of about 2 cm, in which other photoelectric cells are placed, which record how many times the animal places its head inside the hole. This test was performed in the BL condition and on day 7, 14, and 21 after surgery.

### 2.5. Evaluation of anxiolytic-like effect

#### 2.5.1. Open field test

This is a test used to assess the animal's anxiety-like behavior. A rectangular box (78 × 60 × 39 cm) is used, on which an internal perimeter is traced such as to deviate about 3 cm from the walls. Animals were positioned in the center of the box, and then, the time each animal remains in the internal portion is measured, over a total duration of 5 minutes. The longer the animal stays in the center of the arena, the less anxious-like it is. This test was performed in the BL condition and on day 7, 14, and 21 after surgery.^39^

#### 2.5.2. Light-dark box

The light-dark box (LDB) test was performed as previously reported.^8^ Briefly, each mouse was allowed to move freely for 5 minutes in a box with 2 different compartments, the dark and the light chamber, separated by a small door (10 cm × 3.2 cm). The time spent in the light was used as a sign of the anxiety state of each animal. This test was performed at day 28 after surgery.

### 2.6. Evaluation of antidepressant activity

#### 2.6.1. Sucrose splash test

This test is aimed at assessing the level of depression of the mouse. A 10% sucrose solution in H_2_O is prepared, and a small amount of this is placed on the animal's back. The mouse is placed inside a box, and the time it has spent cleaning is measured compared with the total duration of the test 5 minutes. The purpose is to obtain information about the state of depressive-like behavior of the mouse; in fact, the more this will be marked, the more difficult the animal will tend to clean itself. This test was performed in the BL condition and on day 7, 14, and 21 after surgery.^24^

#### 2.6.2. Tail suspension test

The tail suspension test was performed as described by Borgonetti et al.^10^ Mice were suspended from a pole mounted 50 cm above the floor by means of adhesive tape placed in the middle of the tail. The time during which the mice remained immobile was measured with a stopwatch during a 6-minute test period. Mice were considered immobile when they hung passively and completely still, except for movements caused by breathing. An immobile posture (defined as immobility) was considered as a depression-like behavior (behavioral despair) and was measured in the first 2 minutes of the test, when the animals are reacting to unavoidable stress, and in the last 4 minutes, when behavioral despair is established. This test was performed at day 28 after surgery.

### 2.7. Evaluation of mnemonic functions

#### 2.7.1. Novel object recognition test

Memory-related responses were measured using the novel object recognition test (NORT), which is based on the natural exploratory activity of mice. To perform the NORT, an arena was used in which the animal was allowed to move and settle freely. No objects were placed in the box during the setting session. Then, in the first session (day 1: training session), animals were placed in the center of the arena and presented with 2 identical objects (A1 and A2), placed 16 cm from the wall and 37 cm apart, for 5 minutes. Exploration time assessed as sniffing or touching the object with the nose and mouth was then recorded. Two different operators recorded the time of the animal on each object. To measure long-term memory, animals were placed back in the same arena 24 hours after (day 2: retention session) the first test with 2 objects, the familiar A1 (the same as the day before) and a new object B for 5 minutes, placed at the same distance as the first day. To fix the objects in position, Velcro was used in the base of the objects. Objects A1 and B had different shapes, colors, and sizes that had no meaning for the animals. The objects and apparatus were cleaned with an ethanol solution between tests to remove olfactory cues.

From the test, it was noted how long the animals observed the 2 objects A1 and A2, whether there was a difference in discrimination between object A1 and object B, and finally, the percentage of time spent by each animal in exploring the familiar object between the training session (0 hours) and the retention session (24 hours) calculated as: TA1 × 100/5 minutes.^18^ This test was performed at day 28 after surgery.

### 2.8. BV2

A murine immortalized microglial cells (BV2) (mouse, C57BL/6 Tema Ricerca, Genova, Italy), was used for this study. The cells were thawed and placed in a 75 cm^2^ flask (Sarstedt, Milan) in a medium containing RPMI with the addition of 10% heat-inactivated (56°C, 30 minutes) fetal bovine serum (FBS, Gibco, Milan, Italy), 1% glutamine, and 1% penicillin-streptomycin solution. Cells were grown at 37°C and 5% CO_2_ with daily medium change.^7^ To optimize of the senescent model, we used bacterial lipopolysaccharide from Gram (LPS, *Salmonella enteritidis*, Sigma-Aldrich, Milan, Italy) at different concentrations: 100, 250, and 500 ng/mL. A schematic representation of the microglia senescent model has been reported in Table 2.

**Table 2:** Schematic representation of the BV2 senescent model.

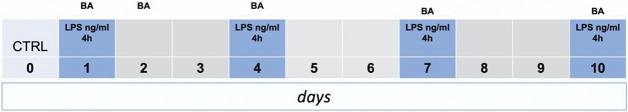

LPS(lipopolysaccharide) has been used at the concentration of 100, 250, and 500 ng/mL.

BA, biological analysis; CTRL, control.

### 2.9. Sulforhodamine B assay

Cell viability was assessed by sulforhodamine B (SRB) assay. Cells (2 × 10^4^ cells in 200 μL) were seeded in 96-well plates, each well corresponding to a different treatment. One hundred microliters of serum-free medium was added to the wells, followed by 25 μL of 50% trichloroacetic acid (TCA). The plate was then incubated at 4°C for 1 hour. Wells were then washed 5 times with double distilled water (100 μL per well). The plate was left overnight to dry at room temperature (rt). The next day, 30 µL of a solution of SRB 4 mg/mL in 1% acetic acid was added to each well and incubated for 30 minutes at rt. Four washes with 1% acetic acid (200 μL per well) were performed, and 200 μL of TRIS HCl solution (pH 10) were added to the wells and incubated for 5 minutes with shaking. Finally, the absorbance was read at a wavelength of 570 nm. The treatments were performed in 6 replicates in 3 independent experiments, and cell viability was calculated by normalizing the values to the control's mean.^50^

### 2.10. Senescence-associated heterochromatin foci analysis

Cells (1 × 10^4^ cells in 500 μL of medium with 3% FBS per well) were initially seeded in 24-well plates, containing previously sterilized slides at the bottom of the wells. After appropriate treatments, the medium was removed, and paraformaldehyde (PFA) 4% was added and incubated for 30 minutes at rt. Wells were washed with phosphate buffer saline (PBS) and then 0.1% PBS-Triton solution for 5 minutes. After 3 washes with PBS, the slides were sealed with a drop of a 1 μg/mL DAPI solution in the mounting medium (90% glycerol + PBS), allowed to dry in the dark at room temperature overnight, and then stored at 20°C. After about a week, slides were observed with an OLYMPUS BX63F fluorescence microscope connected to a PC with an image acquisition card.^52^

### 2.11. Protein extraction

To examine protein expression, mice were killed, and tissues were removed after 7, 14, 21, and 28 days from surgery. Samples were homogenized in a lysis buffer containing 25 mM Tris-HCl pH (7.5), 25 mM NaCl, 5 mM EGTA, 2.5 mM EDTA, 2 mM NaPP, 4 mM PNFF, 1 mM di Na3VO4, 1 mM PMSF, 20 μg/mL leupeptin, 50 μg/mL aprotinin, and 0.1% SDS (Sigma-Aldrich). The homogenate was centrifuged at 12,000*g* for 30 minutes at 4°C, and the pellet was discarded. Proteins from cells were extracted by RIPA buffer (50 mM Tris-HCl pH 7.4, 150 mM NaCl 1% sodium deoxycholate, 1% Triton X-100, and 2 mM PMSF) (Sigma-Aldrich), and the insoluble pellet was separated by centrifugation (12,000*g* for 30 minutes, 4°C). The total protein concentration in the supernatant was measured using the Bradford colorimetric method (Sigma-Aldrich).^41^

### 2.12. Western blotting analysis

Protein samples (30 µg of protein/sample) were separated by 10% SDS-polyacrylamide gel electrophoresis (SDS-PAGE). Proteins were then blotted onto nitrocellulose membranes (90 minutes at 110 V) using standard procedures. Membranes were blocked in PBST (PBS with 0.1% Tween) containing 5% nonfat dry milk for 90 minutes and incubated overnight at 4°C with primary antibodies: anti-β-galactosidase (Santa Cruz Biotechnology Cat# sc-65670, RRID:AB_831022IBA1), (1:1000; Santa Cruz Biotechnology, Santa Cruz, CA, Cat# sc-32725; RRID:AB_667733), anti-p38 MAPK antibody (Cell Signaling Technology, Danvers, MA, Cat# 9212, RRID:AB_330713), anti-I kappa B alpha (Santa Cruz Biotechnology Cat# sc-1643, RRID:AB_627772), and anti-IL6 (Abcam, Cambridge, United Kingdom Cat# ab6672, RRID:AB_2127460). The day after, blots were rinsed 3 times with phosphate-buffered saline with Tween 20 (BPST) and incubated for 2 hours at room temperature with HRP-conjugated secondary antibodies and then detected by the chemiluminescence detection system (Life Technologies Italia, Monza, Italy). Signal intensity (pixels/mm^2^) was quantified using ImageJ (National Institutes of Health, NIH). The signal intensity was normalized to that of GAPDH (1:5000; Santa Cruz Biotechnology).^23^

### 2.13. Immunofluorescence analysis

For immunofluorescence analysis, biological samples have been fixed in formalin at 4% for 24 hours, dehydrated in alcohol, included in paraffin, and finally cut into 20 μm sections of the spinal cord and hippocampus. Primary antibodies used were anti-β-galactosidase (Santa Cruz Biotechnology Cat# sc-65,670, RRID:AB_831022IBA1) and anti-CD11b (Bioss bs-11127; RRID:AB_10856024), added, and diluted 1:100 in 1% BSA in PBS overnight at 4°C. After rinsing in PBS containing 0.01% Triton-X-100, sections were incubated in secondary antibodies Invitrogen Alexa Fluor 488 (490-525, 1:400; RRID:AB_221544), Invitrogen Alexa Fluor 568 (578-603, 1:400; RRID:AB_2534072) (Thermo Fisher Scientific, Waltham, MA), and Cruz Fluor 594 (592-614, 1:400; RRID:AB_2847914) (Santa Cruz Biotechnology) at room temperature for 2 hours. Sections were cover slipped using the Vectorshield mounting medium (Vector Laboratories, Burlingame, CA). A Leica DM6000B fluorescence microscope equipped with a DFC350FX digital camera with appropriate excitation and emission filters for each fluorophore was used to acquire representative images. The immunofluorescence intensity was calculated using ImageJ. Colocalization of 2 different labels was measured using EzColocalization plugin (ImageJ). The extent of colocalization was determined by calculating the Pearson correlation coefficient (PCC). The PCC quantifies the correlation between individual fluorophores considering their intensities. The PCC is characterized by the determined value range: 21, which indicates anticolocalization; 11, which indicates colocalization; and 0, which indicates that there is no colocalization.^4^

### 2.14. Statistical analysis

The data and statistical analysis in this study comply with the recommendation on experimental design and analysis in pharmacology.^14^ The data are presented as mean ± SEM. Results are expressed as mean ± SEM. Repeated measures two-way analysis of variance followed by the Tukey post hoc test was used to compare locomotor behavior, pain behaviors, and mood behavior between neuropathic and sham mice (CTRL). One-way and two-way analyses of variance followed by specific post hoc were used for statistical analysis of in vitro data (n = 6). A Student *t* test was used when necessary. For each test, a value of *P* < 0.05 was considered significant. The software GraphPad Prism version 9.1.2 (GraphPad Software, San Diego, CA) was used in all statistical analyses.

## 3. Results

### 3.1. Nociceptive and emotional behavioral phenotype of spared nerve injury mice

#### 3.1.1. Time course of neuropathic pain development and locomotor dysfunction in spared nerve injury mice

**Figure 1.**
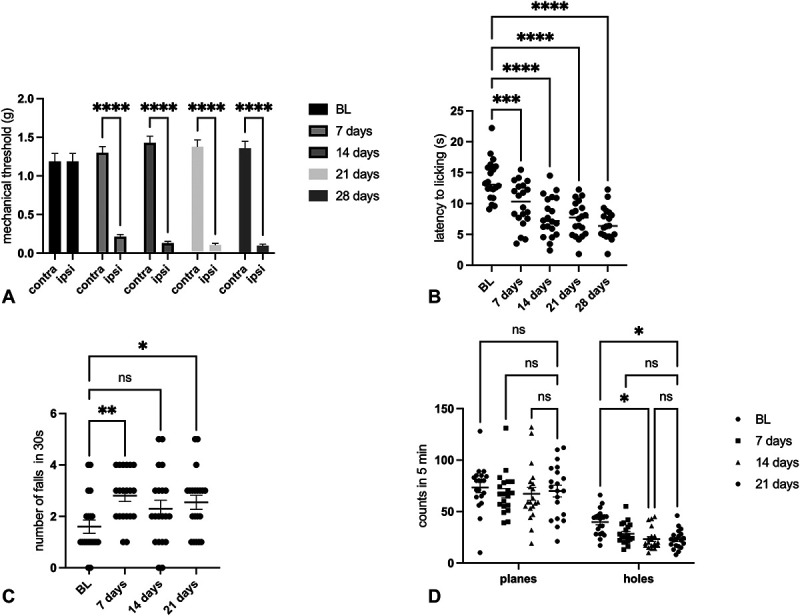
Time course of (A) mechanical allodynia (Von Frey filaments; BL c/i: 1.19 ± 0.10/1.19 ± 0.10; 7 days c/i: 1300 ± 0.08/0.214 ± 0.03; 14 days c/i: 1.43 ± 0.085/0.13 ± 0.023; and 21 days c/i: 1.38 ± 0.08/0.10 ± 0.02), (B) thermal hyperalgesia (hot-plate test; BL: 13.78 ± 0.72; 7 days: 9.94 ± 0.79; 14 days: 7.8 ± 0.72; and 21 days: 7.47 ± 062), (C) locomotor activity (rotarod test; BL: 1.600 ± 0.25; 7 days: 2.80 ± 0.22; 14 days: 2.3 ± 0.32; and 21 days: 2.55 ± 0.27), and (D) spontaneous mobility and exploratory activity (hole-board test planes; BL: 73.45 ± 5.08; 7 days: 67.90 ± 4.54; 14 days: 67.10 ± 6.15; and 21 days: 69.95 ± 5.77; holes: BL: 39.8 ± 2.76; 7 days: 28.35 ± 2.26; 14 days: 23.20 ± 2.30; and 21 days: 22.90 ± 2.03) of SNI mice (n = 25) at 7, 14, and 21 days after surgery. One-way ANOVA ****P < 0.0001, **P < 0.01, and *P < 0.05. ANOVA, analysis of variance; BL, baseline; ns, not significant; SNI, spared nerve injury.

The first aim of this work is to characterize the phenotype of SNI animals and then associate it with senescence-related mechanisms. To confirm that animals with neuropathy in the used cohort developed the symptoms associated with NP, we assessed mechanical allodynia, thermal hyperalgesia, and locomotor activity once a week. Spared nerve injury mice developed severe mechanical allodynia (Fig. 1A) and thermal hyperalgesia (Fig. 1B) as early as 7 days after surgery that persisted on days 14, 21, and 28 after nerve injury. The animals also developed motor impairment, measured by the rotarod test, as the SNI animals fell more times from the rotating rod in 30 seconds than control animals, as early as 7 days after the operation. Animals appeared to have a slight recovery after 14 days but then worsen again after 21 days (Fig. 1C). Locomotor activity was also assessed using the hole-board test, in which both spontaneous activity (planes) and exploratory function (holes) are considered. Spared nerve injury animals' spontaneous mobility did not change, differently from exploratory ability that was significantly reduced (Fig. 1D).

#### 3.1.2. Assessment of appearance of anxiety-like symptoms in animals with neuropathy

**Figure 2.**
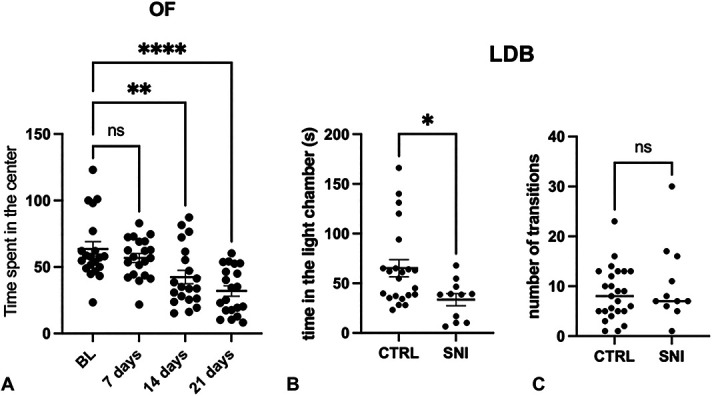
Time course of anxiety-like symptoms in SNI mice measured with the (A) open field test (OF; BL: 63.62 ± 5.40; 7 days: 56.79 ± 3.34; 14 days: 42.49 ± 5.01; and 21 days: 32.01 ± 3.90). One-way ANOVA ****P < 0.0001, **P < 0.01. In the light-dark box (LDB) test, we measured the time in the light chamber (B; CTRL: 65.18 ± 8.53; SNI: 33.53 ± 6.029) and the number of transitions (C; CTRL: 8.50 ± 1.05; SNI: 10.45 ± 2.41) 28 days after surgery. Student *t* test *P < 0.05. ANOVA, analysis of variance; BL, baseline; CTRL, control; LDB, light-dark box; ns, not significant; SNI, spared nerve injury.

To evaluate the occurrence of anxiety-like behavior in animals, the open field test was performed and repeated once a week for 4 weeks. The open field is one of the few tests that can be repeated on the same animal for the control of anxiety because, in this test, the animal assumes a completely spontaneous behavior. In fact, in this test, the animals are placed in a box with walls, and the time that the animals remain in the center of the box is registered as an indication of the state of anxiety of the animals. The SNI animals developed anxiety starting from day 14 after surgery, as shown by the reduction of the time spent in the center of the box, compared with time 0 (BL). This type of behavior was maintained until day 21 after surgery (Fig. 2A). On day 28 after the operation, we further confirmed the anxious symptomatology of the animals by means of the LDB test, a widely used and largely accepted test to evaluate the presence of an anxiety-like behavior by measuring the time animals spent in the light vs dark part of the box and the number of transitions made to switch between compartments. Spared nerve injury animals spent less time in the light than control animals, confirming an anxious-like state in operated vs sham-operated animals (Fig. 2B). By contrast, SNI did not appear to alter the number of transitions (Fig. 2C).

#### 3.1.3. Evaluation of the appearance of similar depressive symptomatology in animals with neuropathy

**Figure 3.**
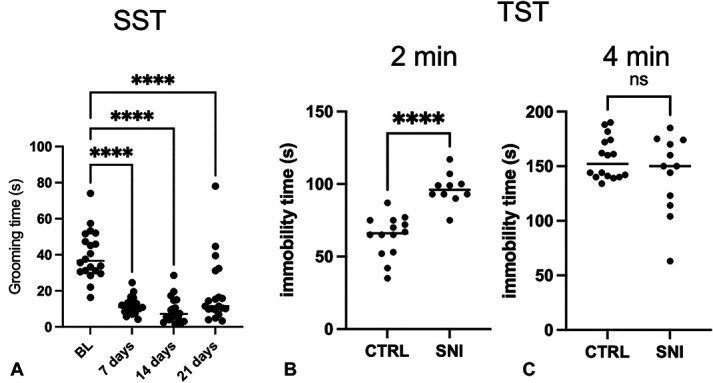
Time course of depression-like symptoms in SNI mice measured with (A; BL: 39.84 ± 3.04; 7 days: 11.64 ± 1.10; 14 days: 9.16 ± 1.53; and 21 days: 18.97 ± 4.06) the sucrose splash test (SST) at BL, 7, 14, and 21 days from surgery. One-way ANOVA ****P < 0.0001. After 28 days from surgery, we performed the tail suspension test (TST), registering the immobility time at 2 minutes (B; CTRL: 55.71 ± 3.80; SNI: 23.40 ± 3.49) and 4 minutes (C; CTRL: 82.96 ± 4.77; SNI: 97.33 ± 10.31). Student *t* test ****P<0.0001ANOVA, analysis of variance; BL, baseline; CTRL, control; ns, not significant; SNI, spared nerve injury.

Aside with the evaluation of anxiety-like symptoms, we also assessed the occurrence of symptoms associated with depression. To evaluate the appearance of depression, we performed once a week the sucrose splash test, a test in which the animal is placed inside a box and wet on the back with a 10% sucrose solution. The test has a total duration of 5 minutes, during which the time spent by the animal cleaning itself is measured, which will be inversely proportional to its depressive state. This is because, in a state of tranquility, the mouse will tend to take more care of its appearance. Spared nerve injury animals seem to develop depression-like symptomatology as early as 7 days after surgery, which persisted at 14 and 21 days (Fig. 3A). At day 28, we performed the tail suspension test, a widely used and predictive animal test. The immobility time was measured in the first 2 minutes of the test, in which the animal reacts towards the aversive condition and in the next 4 minutes, in which a behavioral despair arises and the animal no longer react to escape the aversive condition. As shown in Figure 3B, SNI animals have a basal depressive-like symptomatology, as shown by the higher immobility time, while in the following 4 minutes, there is no significant difference with the control animals (Fig. 3C).

#### 3.1.4. Effect of the operation on the mnemonic state of spared nerve injury animals

**Figure 4.**
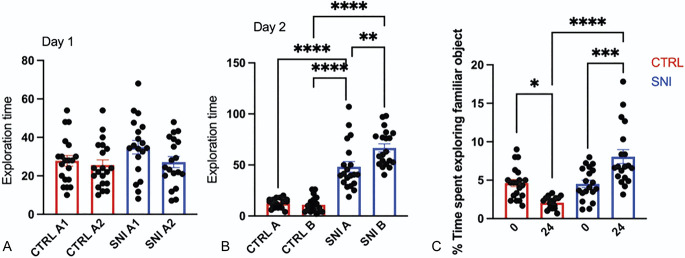
Evaluation of cognitive impairment in SNI mice measured with the novel object recognition test (NORT) after 28 days from surgery. The parameters that we used are the exploration time at day 1 (A; CTRL A1: 27.80 ± 2.73, A2: 25.60 ± 2.67; SNI A1: 34.92 ± 3.52, A2: 27.13 ± 2.86) and after 24 hours (B; day 2; CTRL A: 12.47 ± 1.13, B: 25.11 ± 1.80; SNI A: 48.26 ± 5.16, B: 26.72 ± 3.91). Comparison of (C; CTRL 0: 4.63 ± 2.04, 24: 2.07 ± 0.18; SNI 0: 4.52 ± 0.47, 24: 8.06 ± 0.90) the time spent exploring the familiar object between CTRL and SNI mice. Two-way ANOVA ****P < 0.0001, ***P < 0.001, and **P < 0.01. ANOVA, analysis of variance; CTRL, control; SNI, spared nerve injury.

The alteration of memory capacity was assessed using the NORT after 28 days from surgery compared with the sham-operated group (CTRL).^10^ In the training session of the NORT (internal control), the total time spent exploring both objects by SNI mice was comparable with that of the control mice group (Fig. 4A). In the retention session (24 hours after the first session), the evaluation of the exploration times between the training object and the novel object illustrated that SNI treatment increased novel object (Fig. 4B) and familiar object (Fig. 4C) exploration time, compared with control mice, suggesting a mnemonic alteration.

### 3.2. Microglial cellular senescence in the spinal cord and hippocampus of spared nerve injury mice

Microglia, when subjected to continuous harmful stimuli, tend to modify their morphology and activity, manifesting a hyperactivation related to increased expression of inflammatory factors that in the long term can damage the surrounding neuronal bodies, thus promoting the onset of neurodegenerative diseases.^1^ As senescence and microglial activation seems to be involved in the physiopathology of both anxiety and pain, we investigated these conditions in the spinal cord and hippocampus. The main marker of senescence is the β-galactosidase (β-gal), whose increase in the CNS is a sign of cellular aging.

**Figure 5.**
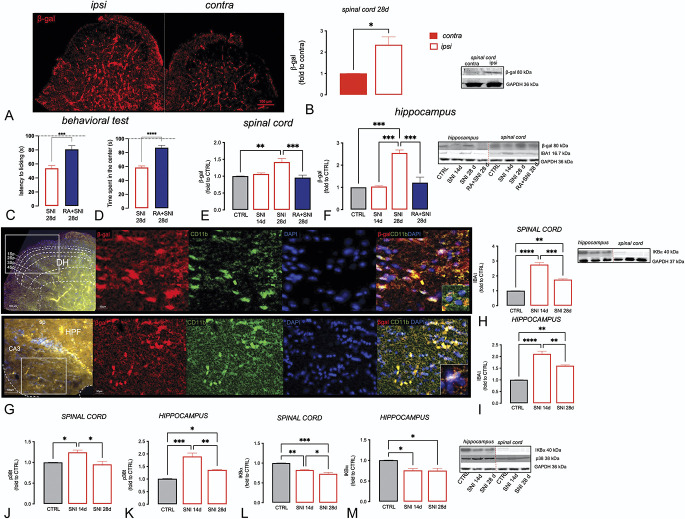
Evaluation of cellular senescence markers in the spinal cord and hippocampus of SNI mice: Expression (A) and quantification (B) of β-gal in spinal cord samples on day 28 after surgery. Attenuation of the nociceptive (C) and anxiety-related (D) phenotype and reversal of β-gal overexpression in the spinal cord (E) and hippocampus (F) by rosmarinic acid (RA, 5 mg/kg p.o.), used as the reference drug. (G) Immunofluorescence images of the spinal cord dorsal horn (upper) and hippocampus (bottom) of SNI mice (n = 6). Scale bar 200 µm and 50 µm. Quantification of IBA1 (H, spinal cord; I, hippocampus), p38T (J, spinal cord; K, hippocampus), and Iκ-Bα (L, spinal cord; M, hippocampus) of CTRL (n = 6), SNI mice 14 days (n = 6), and SNI mice 28 days (n = 6) after surgery. Representative blots were reported. One-way ANOVA ****P < 0.0001, ***P < 0.001, **P < 0.01, and *P < 0.05. ANOVA, analysis of variance; CTRL, control; DH, dorsal horn; HPF, hippocampal formation; SNI, spared nerve injury.

The SNI animals showed an increased expression of β-gal at the level of the ipsilateral spinal dorsal horn (relative to the injured side) compared with the contralateral (relative to the uninjured side), as shown for immunofluorescence (Fig. 5A) and Western blotting assay (Fig. 5B). This effect was observed 28 days from the operation, when neuropathic pain and neuropathy-associated symptoms were well established, leading us to hypothesize that the lesion is involved in cellular senescence processes. To support the hypothesis of a correlation between senescence and SNI-associated symptoms, SNI mice were treated with rosmarinic acid (RA5; mg/kg p.o.), used as the senomorphic reference drug,^6^ that showed antihyperalgesic (Fig. 5C) and anxiolytic activity (Fig. 5D). Furthermore, RA treatment reduced β-gal expression both in the spinal cord (Fig. 5E) and hippocampus (Fig. 5F). Of note, no alteration in the β-gal expression compared with the CTRL group was observed 14 days after surgery.

To verify the expression of microglia senescent cells, we measured the colocalization between CD11b, a well-known marker of microglia (green), and β-gal (red) in the spinal cord and hippocampus (Fig. 5G; Supplementary Figure S1, available at http://links.lww.com/PAIN/B738). On day 28, we observed that CD11b and β-gal colocalized in the dorsal horn of SNI mice with a Pearson correlation coefficient (PCC) of 0.73 ± 0.04, whereas in the hippocampus the PCC value was 0.83 ± 0.020, highlighting a high level of colocalization. Consistently with previous data, an increase of IBA1 expression has been detected in the spinal cord (Fig. 5H) and hippocampus (Fig. 5I) of SNI mice, highlighting the presence of microgliosis in coincidence with neuropathy-related symptoms. The peak of IBA1 expression has been observed 14 days after surgery, with a trend to decrease after 28 days. To further confirm the role of microglia, 2 additional markers of proinflammatory microglia were evaluated, p38 and Nuclear Factor-κB (NF-κB), that represent early and late factors of senescent-associated secretory phenotype (SASP), respectively. P38 followed the same time course of IBA1 expression, with a peak of the protein expression after 14 days and then diminishing 28 days after surgery, both in the spinal cord (Fig. 5J) and hippocampus (Fig. 5K). The expression of the NF-κB inhibitory protein IκB was reduced with a comparable intensity 14 and 28 days after surgery in the spinal cord tissue (Fig. 5L) and hippocampus (Fig. 5M), indicating a prolonged activation of the NF-κB pathway. From these analyses, we summarized that, 28 days after surgery, the microgliosis persisted, and there was an increase in the senescence marker expression in both the hippocampus and the spinal cord.

### 3.3. Evaluation of cellular mechanism of microglial senescence in BV2 cells

**Figure 6.**
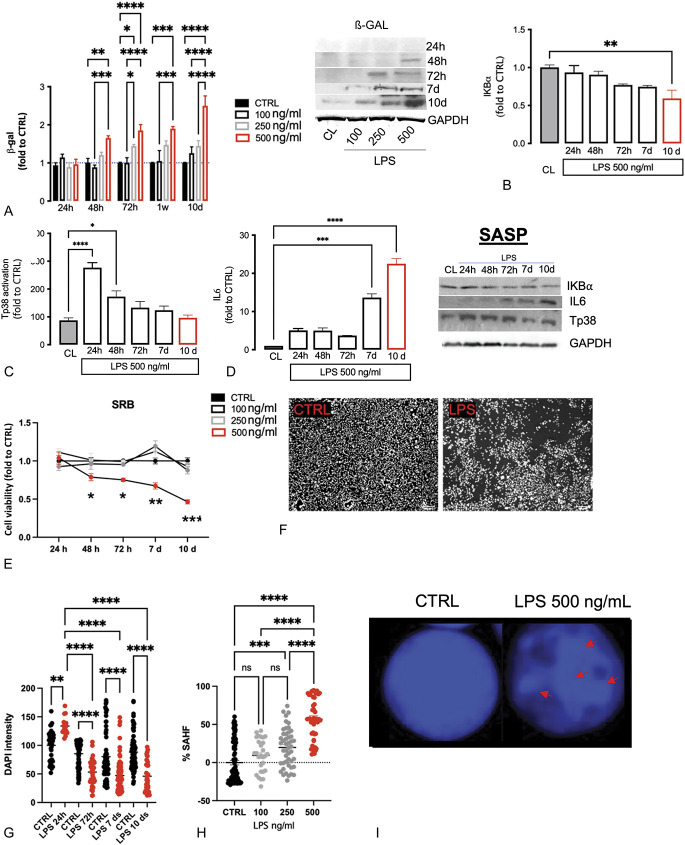
Evaluation of cellular and molecular alteration in BV2 senescent cells: (A) Time course of β-gal protein expression in BV2 stimulated with LPS at 100, 250, and 500 ng/mL. Time course of IKB⍺ (B), Tp38 (C), and IL-6 (D) protein expression in BV2 intermitting stimulated with LPS at 500 ng/mL. (E) Cell viability test measured with SRB was observed at 24 hours, 48 hours, 72 hours, 7 days, and 10 days in LPS-stimulated BV2 at the concentration of 100, 250, and 500 ng/mL. (F) Representative images of LPS-stimulated (500 ng/mL) BV2 at day 10 compared with the CTRL (scale bar 100 μm). (G) Time course of DAPI intensity in LPS-stimulated (500 ng/mL) BV2 and (H) the percentage of foci expressed in cells. (I) Representative images of SAHF in LPS-stimulated BV2 after 10 days (scale bar 20 μm). One-way and two-way ANOVA; ****P < 0.0001, ***P < 0.001, **P < 0.01, and *P < 0.05. Representative blots are also showed. ANOVA, analysis of variance; β-gal, β-galactosidase; CTRL, control; SAHF, senescence-associated heterochromatin foci; SASP, senescent-associated secretory phenotype; SRB, sulforhodamine B.

To define the expression pattern of senescence markers in microglia more selectively and to correlate these events with those observed in neuropathic mice tissue, we used an in vitro microglial senescence model. Murine microglial BV2 cells were stimulated with 3 different concentrations of LPS (100, 250, and 500 ng/mL) for 4 hours per day, at various time intervals (24, 48, and 72 hours, 7 and 10 days), over a total period of 10 days. Several factors were considered to select the optimal combination: expression of β-gal (Fig. 6A), expression of factors related to senescent-associated secretory phenotype (SASP) (Figs. 6B–D), cell viability (Fig. 6E) and morphology (Fig. 6F), and development of nuclear senescence-associated heterochromatin foci (SAHF) (Figs. 6G–I; Supplementary Figure S2, available at http://links.lww.com/PAIN/B738).

β-gal is a major marker associated with cellular aging. LPS 100 ng/mL did not alter β-gal levels at any time point. LPS 250 ng/mL induces a significant increase after 72 hours, which became stationary after 7 and 10 days. LPS 500 ng/mL, starting from 48 hours, altered β-gal expression, which increased over time, reaching a maximum concentration after 10 days (Fig. 6A).

As observed above, senescent cells can interact with the environment through the release of several factors with various pleiotropic effects. These are known as SASP.^32,52^ NF-κB is part of these bioactive elements. By evaluating NF-κB activation through dosing the levels of the inhibitory protein IκB⍺, we found that LPS 500 ng/mL led to a gradual decrease in IκB⍺ inhibitor over time, with the result of being most pronounced after 10 days (Fig. 6B). As observed in the tissue samples, IκB⍺ appeared to represent a late SASP marker (Fig. 6B), whereas p38, peaking 24 hours after stimulation and then gradually declining over time, represented an early SASP indicator (Fig. 6C). Cytokines are a sign of late SASP that correlates directly with NF-κB activation, and in this model, the maximal release of IL-6 started after 7 days and peaked after 10 days from the beginning of the pattern, similarly to what observed for IκB⍺ (Fig. 6D).

Cell viability is another parameter to be considered because senescent cells undergo cell death more rapidly. Through the SRB test, we found that LPS at concentrations of 100 and 250 ng/mL did not significantly alter microglia survival, compared with untreated controls. The concentration of 500 ng/mL produced a significant reduction in cell viability starting from 48 hours, with more evident effects after 7 and 10 days (Fig. 6E). Figures 6G–I showed the results obtained using the SAHF immunofluorescence technique, which highlighted the development of senescence foci at the nuclear level. The nucleus of untreated cells was homogeneous in shape and density, whereas the nucleus of LPS-stimulated (500 ng/mL) cells was more fragmented, with evident gaps corresponding to foci of senescence, a symptom of an ongoing process of cellular aging (Figs. 6G and I). Other concentrations of LPS produced a dose-dependent effect with LPS 100 ng/mL ineffective and LPS 250 ng/mL only partially effective. Treating cells with LPS 500 ng/mL produced a progressive increase over time in the percentage of foci at the level of the nucleus. An important increase in the foci occurs after 7 and 10 days, which is consistent with the other parameters of senescence evaluated (Fig. 6H).

## 4. Discussion

In this work, we primarily focused on identifying and characterizing the occurrence of anxiety, depression, and impaired cognitive abilities in a model of peripheral neuropathy and to correlate these pain-associated symptoms with microglia senescence.

In a neuropathic condition, the reduction of pain of at least 30% after treatment is considered as a clinically significant result.^12^ However, most patients do not show sufficient relief after treatment, and this is due to the heterogeneity of the mechanisms underlying the pathological situation and the coexisting emotional and psychological aspects. Thus, an aspect to be considered is the effectiveness of the treatment undertaken compared with comorbidities associated with pain, such as mood disorders and sleep alterations, conditions that are often debilitating.^38^ Indeed, the presence of depression and anxiety may represent an obstacle in the therapy of pain, and these conditions should be identified and characterized to develop specific treatment strategies.^35^

Changes in plasticity at the central level induced by SNI have been described to promote the onset and development of the negative psychological components associated with a NP condition, such as anxiety, depression, and aggressive behavior.^20^ The possible occurrence of mood disorders has been observed also in other models of NP. Animals operated to develop chronic constriction injury (CCI) have been used as a model for the study of anxiety, depression, and mnemonic alterations as behaviors related to NP.^16^ Similarly, the partial sciatic nerve ligation (pSNL) model is also used to assess the development of mood disorders associated with NP.^48^ Consistent with these findings, a temporal analysis of the development of pain and pain-associated symptoms showed that SNI mice developed a persistent and prolonged mechanical allodynia and thermal hyperalgesia in the first days after injury, followed by the appearance of NP-associated anxiety and depression 14 days after surgery, which becomes increasingly stronger after 28 days. It has been observed in clinical trials that patients with chronic pain also developed a cognitive deficit in most cases.^34^ In agreement with clinical reports, cognitive impairment was also observed in SNI animals 28 days after the operation, using the NORT.

The well-known role of microglia in the promotion of NP encouraged us to investigate their role also in pain-associated behavioral symptoms. Hyperactivation of microglia with high levels of proinflammatory cytokines was detected in multiple brain regions during psychiatric pathological conditions.^21,25,44,45^ Indeed, microglia play a key role in synaptic remodeling not only in the postnatal phase but also in adulthood by influencing synapse stability in neuronal circuits.^51^ Consistent with these observations, we detected microglia activation within both the spinal cord and hippocampus, 2 distinct areas with a key role in pain and mood disorders, respectively. The activation of microglia toward a proinflammatory phenotype can compromise neuronal integrity, because of the overexpression of inflammatory cytokines and oxygen free radicals (ROS) and decreased levels of neuroprotective factors.^13^ However, as occurs in many pathological contexts, the inflammatory process tends to become chronic because of excessive microglial activation, with acceleration of the aging process and loss of function.^37^ Indeed, a typical feature of aging is a chronic low-grade inflammation that is associated with a high level of circulating cytokines and other inflammatory markers, such as coagulation factors. The age-related increment of proinflammatory markers induces a cellular response consisting of an increased level of anti-inflammatory molecules.^17,33^ Excessive proinflammatory stimulation and/or an inefficient anti-inflammatory response cause disability/frailty and the onset of age-related pathological conditions.

Thus, we investigated the presence of microglial senescent cells and their correlation with pain-associated comorbidities. On a cellular level, aging is accompanied by the accumulation of cells that adopt a specific phenotype, known as senescence. Cellular senescence is defined as a cell fate in which proliferating or differentiated cells undergo replication arrest. Even if these cells stop dividing, they are metabolically active and secrete a plethora of proinflammatory cytokines and mediators, collectively termed the senescence-associated secretory phenotype (SASP).^37^ Over time, senescent cells accumulate in all tissues and organs, contributing to their functional deterioration.^26^ Consistently, in our model, we observed an increase in the expression of β-gal, a marker of cellular aging, in microglia cells at the level of the spinal cord and hippocampus, 28 days after surgery. In both neuronal areas, the increase of the microglial marker IBA1 was detected from prior to β-gal overexpression, and it began to decline after 28 days in coincidence with β-gal increase. In addition, an “early” SASP, such as p38, and a “late” SASP, such as NF-κB, both markers of microglial senescence, were increased after 14 and 28 days, respectively.^40^ Studies on animal models subjected to depression showed that in the hippocampus, but not in other brain regions, there was an initial activation of microglia followed by an apoptotic decline of these cells and a reduction in activity, caused by a reduction in the cell number, about 5 weeks later. Restoration of microglial homeostasis by blockade of the initial activation of hippocampal microglia or promoting microglial proliferation at later stages partially or totally reversed depressive behavior, confirming the role of microglia in depressive pathological conditions.^27^ Data on microglia modification toward a senescent phenotype observed in neuropathic mice were more selectively extended and confirmed by an in vitro model of microglial senescence, which mimics cellular aging induced by a proinflammatory stimulus. An increase of β-gal was observed in BV2 cells after the intermittent stimulation with LPS 500 ng/mL for 10 days, along with an overexpression of markers of early SASP (increase of p38 at 24 hours) and late SASP (increase of IL-6 and NF-κB after 10 days), as well as a progressive reduction of the number of viable cells compared with the unstimulated control. Senescence-associated heterochromatin foci are specialized heterochromatin domains that contribute to the silencing of proliferation-promoting genes, promoting aging.^6,53^ BV2 cells treated with LPS 500 ng/mL for 10 days also showed a much less homogeneous DAPI staining compared with the control, which is consistent with the formation of SAHF.

## 5. Conclusion

Summarizing, 14 days after surgery, in addition to a well-established pain hypersensitivity, we observed the appearance of depressive-like and anxiolytic-like symptoms, along with a strong spinal and hippocampal microglia activation. After 28 days from surgery, while mood symptoms were well established, microglia overexpression was reduced, and contemporary, the appearance of inflammatory-related senescence markers in microglial cells was observed. Our study showed, for the first time, the presence of hippocampal microglia senescence in SNI mice. The hippocampal cellular alterations paralleled those observed at the spinal cord level, further supporting the hypothesis of a correlation between NP-associated comorbidities and microglia senescence. A very recent study showed that 4 months after SNI, mice displayed telomere length reduction and p53-mediated cellular senescence in the spinal microglia cells, which was responsible for the maintenance of pain.^36^ Our results showed microglia alterations from 1 month after surgery. We can, thus, suppose that the proinflammatory senescent microglia phenotype might represent an early pain-associated senescence-related event with a key role in both sensory and emotional alterations. Therefore, present findings could represent an important step to better understand the pathophysiological cellular mechanisms in chronic pain states and related comorbidities.

## Conflict of interest statement

The authors have no conflicts of interest to declare.

## Appendix A. Supplemental digital content

Supplemental digital content associated with this article can be found online at http://links.lww.com/PAIN/B738.

## Supplementary Material

SUPPLEMENTARY MATERIAL
