# Supplementary material for: Microglia senescence is related to neuropathic pain–associated comorbidities in the spared nerve injury model
Source: Pain. 2022 Oct 19;164(5):1106–17. doi: 10.1097/j.pain.0000000000002807 (PMC10108589; doi:10.1097/j.pain.0000000000002807)
Supplement: SUPPLEMENTARY MATERIAL [file jop-164-1106-s001.pdf]

**Supplementary Information**

# Microglia senescence as a potential target in neuropathic pain induced by nerve injury and associated comorbidity.

**Running title:** Microglia senescence as novel target in neuropathic pain

[a]Vittoria Borgonetti, [a]Nicoletta Galeotti

[a]Department of Neuroscience, Psychology, Drug Research and Child Health (NEUROFARBA), Section of Pharmacology and Toxicology, University of Florence, Viale G. Pieraccini 6, 50139 Florence, Italy

Address correspondence to:

Prof Nicoletta Galeotti, Department of Neuroscience, Psychology, Drug Research and Child Health (NEUROFARBA), Section of Pharmacology and Toxicology, University of Florence, Viale G. Pieraccini 6, 50139 Florence, Italy, nicoletta.galeotti@unifi.it

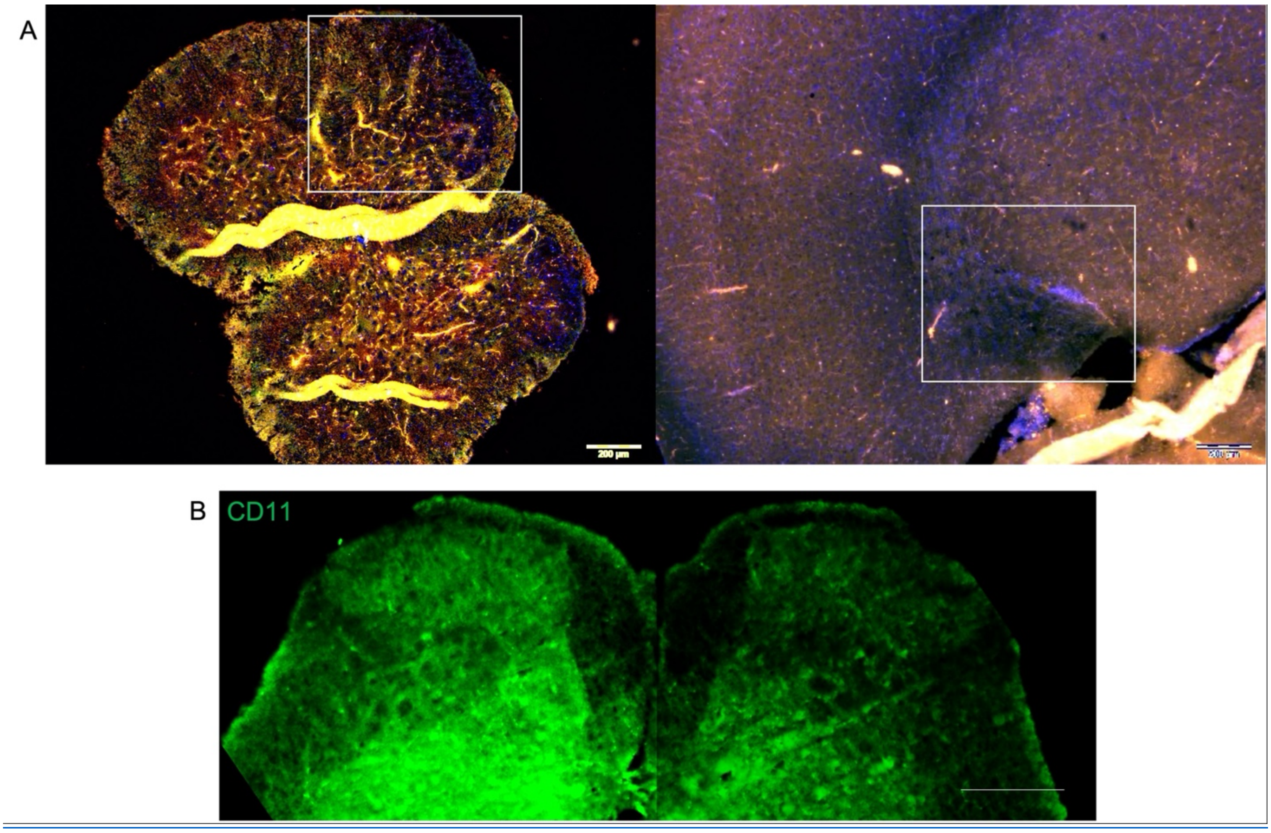


Figure S1. A) Low magnification immunofluorescence micrographs for CD11b staining of the spinal cord and hippocampus. B) Microgliosis in the dorsal horn spinal cord of SNI mice.

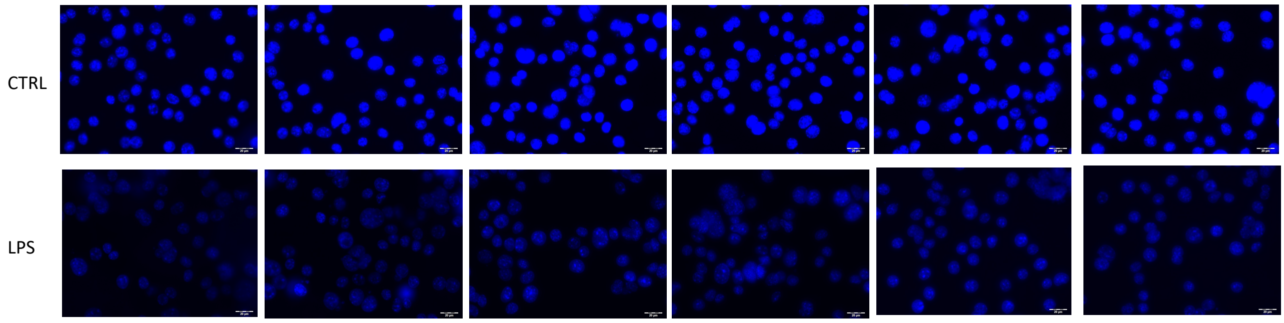


Figure S2. Low magnification images used for DAPI intensity and SAHF assessment in BV2 cells treated with LPS for 4h/day for 10 days (LPS) compared the untreated cells (CTRL).

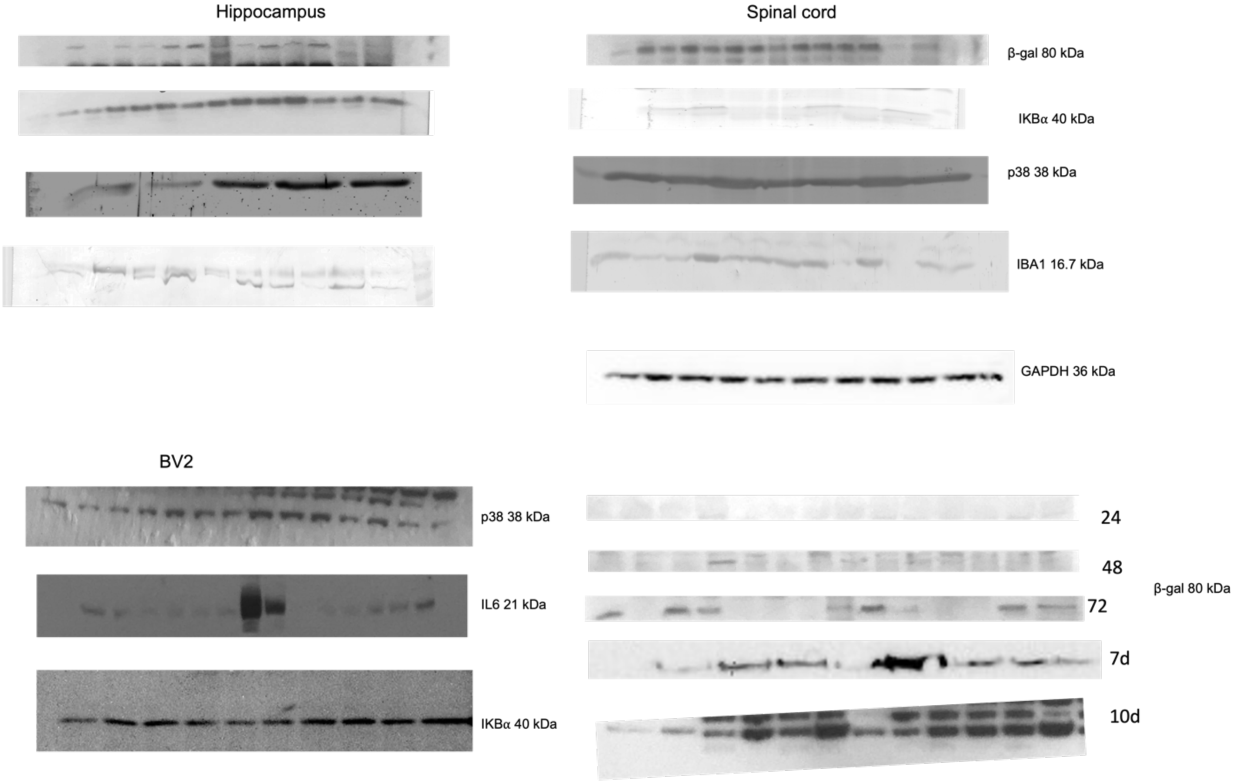


Original blots
